# Reference-agnostic representation and visualization of pan-genomes

**DOI:** 10.1186/s12859-021-04424-w

**Published:** 2021-10-16

**Authors:** Qihua Liang, Stefano Lonardi

**Affiliations:** Department of Computer Science and Engineering, University of California, Riverside, CA 92521 USA

**Keywords:** Comparative genomics, Pan-genome, Genome analysis

## Abstract

**Background:**

The pan-genome of a species is the union of the genes and non-coding sequences present in all individuals (cultivar, accessions, or strains) within that species.

**Results:**

Here we introduce PGV, a reference-agnostic representation of the pan-genome of a species based on the notion of consensus ordering. Our experimental results demonstrate that PGV enables an intuitive, effective and interactive visualization of a pan-genome by providing a genome browser that can elucidate complex structural genomic variations.

**Conclusions:**

The PGV software can be installed via conda or downloaded from https://github.com/ucrbioinfo/PGV. The companion PGV browser at http://pgv.cs.ucr.edu can be tested using example bed tracks available from the GitHub page.

**Supplementary Information:**

The online version contains supplementary material available at 10.1186/s12859-021-04424-w.

## Background

As more and more individuals (cultivars, accessions, or strains) of a given species are sequenced and made available, the adequacy of a single DNA sequence as *the reference* genome for a species is being challenged [1]. Declaring one individual as *the reference* for a species introduces a variety of representational biases in downstream analyses, including SNP discovery, structural analysis, genome-wide association studies, etc. [1]. Instead, the pan-genome captures the entire genetic diversity of a species by cataloging all the structural variants of its genome [2]. According to [3–5] a pan-genome is composed of (i) the *core* genome containing DNA sequences present in all individuals within the species, (ii) the *dispensable* genome containing DNA sequences present in at least two (but not all) individuals,(iii) *unique* (or *private*) genome which includes individual-specific DNA sequences. An effective representation (and its visualization) of a pan-genome is particularly challenging due to the complex rearrangements that can be observed when comparing multiple genomes of the same species [2].

As a testament of this analytic complexity, the majority of the available pan-genome analysis tools either (i) focus only on the genes, or (ii) they can only handle small genomes (e.g., bacterial genomes) and are unable to scale to larger eukaryotic genomes, or (iii) they require users to arbitrarily label one of the individual genomes as *the* reference. For instance, PanX first identifies orthologous gene clusters from a set of individual genomes, then allows users to interactively explore the relationships between genes via a web-based visualization tool [6]. Similarly, PanWeb [7] is a web-based front-end for PGAP (Pan-Genome Analysis Pipeline) [8]. PGAP provides several types of gene-level analysis, including gene cluster analysis, pan-genome profile analysis, variation analysis, evolution analysis and function enrichment analysis. PPanGGOLiN models a microbial pan-genome using a graph in which nodes represent gene families and edges represent genomic neighborhood [9]. The Genome Context Viewer is a genome browser that can identify and visualize micro-synteny regions, i.e., collinear arrangement of homologous genes, in a pan-genome. The Genome Context Viewer allows the exploration of precomputed macro-synteny blocks in pan-genomes [10]. Other tools provide genome-wide insights by comparing whole genomes. PanSeq identifies core, accessory and novel regions of genome-level by carrying out a pairwise alignment against one of the individual genome which need to be considered *the* reference [11]. PGAP-X is an extension of PGAP which uses whole genome sequence alignment to distinguish core, dispensable and strain-specific genes [12]. While PGV and PGAP-X have some common functionalities, PGAP-X does not compute or rely on the consensus ordering. Instead PGAP-X focuses on clustering of gene orthologs. While PGAP-X was designed and extensively tested on bacterial genomes, we were unable to run PGAP-X on large eukaryotic genomes like those used in this study. Table 1 summarizes the main features and limitations for these tools.

**Table 1:** Comparison of pan-genome analysis tools (MSA = multiple sequence alignment)

	Gene based	Genome based	Reference agnostic	Alignment method	Largest genome tested	References
PanX	$\checkmark$			Pairwise alignment using diamond	Microbial genomes	[6]
PGAP	$\checkmark$		$\checkmark$	All-pairs alignment and BLAST-all	Microbial genomes	[8]
PGAP-X	$\checkmark$	$\checkmark$	$\checkmark$	MSA using progressiveMauve	Microbial genomes	[12]
PPanGGOLiN	$\checkmark$			Uses gene families	1000s of microbial genomes	[9]
PanSeq		$\checkmark$		Pairwise alignment	Not mentioned	[11]

In response to the limitations of the existing methods for pan-genome analysis, we propose here a novel pan-genome representation called PGV. The PGV representation is (i) reference-agnostic (i.e., there is no need to artificially declare one of the individual genome to be the reference), (ii) can handle large eukaryotic genomes, and (iii) is very intuitive and simple to understand. The PGV representation can be visualized by a dot-plot or using our PGV genome browser, in which each block is colored depending on whether it is core, dispensable or unique. Structural variations such as inversions and translocations are highlighted, and shared core/dispensable blocks are linked to illustrate how the different accessions relate to each other. PGV performs genome level analyses in which genomic blocks and structural variations are not limited to gene regions. Traditional pan-genome tools are gene centered and often require a reference, while PGV is gene-agnostic and reference-agnostic, thus significantly different outputs are expected from PGV. Users are also allowed to upload annotation tracks (e.g., gene annotations) so that relationships between genes and genome level variations can be identified and visualized. PGV depends on progressiveMauve for the multiple sequence alignment, which is computationally expensive. As a consequence, we expect about a dozen eukaryotic-sized genomes or about two dozen bacterial genomes to be a practical upper-limit to the analysis pipeline.

## Implementation

PGV was implemented using Python3 and a few libraries like numpy, matplotlib, biopython and alignment. PGV can be easily installed via Conda, which takes care of software dependencies and versions. PGV uses matplotlib to generate the dotplots like those shown in Fig. 3. Alternatively PGV generates BED files, which can be visualized by the genome browser at http://pgv.cs.ucr.edu. The genome browser was implemented using Javascript and HTML. The current version is hosted on Google Firebase. The D3.js library was used for data binding. Canvas was used for genome plotting. The Bootstrap library was used for the front-end cosmetics.

The source code for PGV and the genome browser are available at https://github.com/ucrbioinfo/PGV. The github page offers sample data to test the software installation.

## Methods

The input to PGV is a set of *n* individual genomes for the same species, or a set of genomes from very closely-related species. To obtain the best results, input genomes must have a similar level of assembled contiguity. First, PGV carries out a genome-wide multiple sequence alignment using progressiveMauve [13]. Based on the output of the multiple sequence alignment, PGV classifies each alignment block into three types. A *core* genome block, or C-block, corresponds to an alignment that contains all *n* individuals. A *dispensable* genome block (also called *accessory*), or D-block, corresponds to an alignment which contains at least two individuals and at most $n-1$. A *unique* genome block (also called *strain-specific*), or U-block, is a block that belongs exclusively to one individual genome. Please note that in the literature a unique block is a special type of dispensable block, while PGV distinguishes them. Next, PGV converts each individual genome into an ordered sequence of C, D, and U-blocks, each with its corresponding identifier (represented by a unique integer). In the example in Additional file 1: Figure S1(a), the alignment of the five input genomes has produced seven core blocks, four dispensable blocks and nine unique blocks.

After the conversion of each genome into blocks, PGV computes the *consensus ordering* for the C-blocks, which will constitute the “back-bone” of the pan-genome. If we only consider C-blocks, observe that each genome can be represented by a permutation $\sigma$ of the C-block identifiers $\{1, 2, \ldots , m\}$, where *m* is the number of C-blocks. Let $\sigma ^i$ be the permutation for the *i*-th genome, where $i\in [1,n]$. We define the *consensus ordering* of the C-blocks as the ordering $\sigma ^*$ that minimizes the quantity $\sum _{i=1}^n L(\sigma ^i, \sigma ^*)$ where *L* is the Levenshtein (edit) distance between the permutations. In the literature, the string $\sigma ^*$ is called the *median string* of the set $\sigma ^i$. The problems of finding the median for a set of strings under the Levenshtein distance is known to be NP-complete [14]. Similar theoretical results have been derived from more complex metrics [15]. The related notion of consensus ordering for homology blocks was proposed by [16], but their pan-genome is captured by complex bidirectional sequence graphs instead of linear paths.

**Fig. 1:**
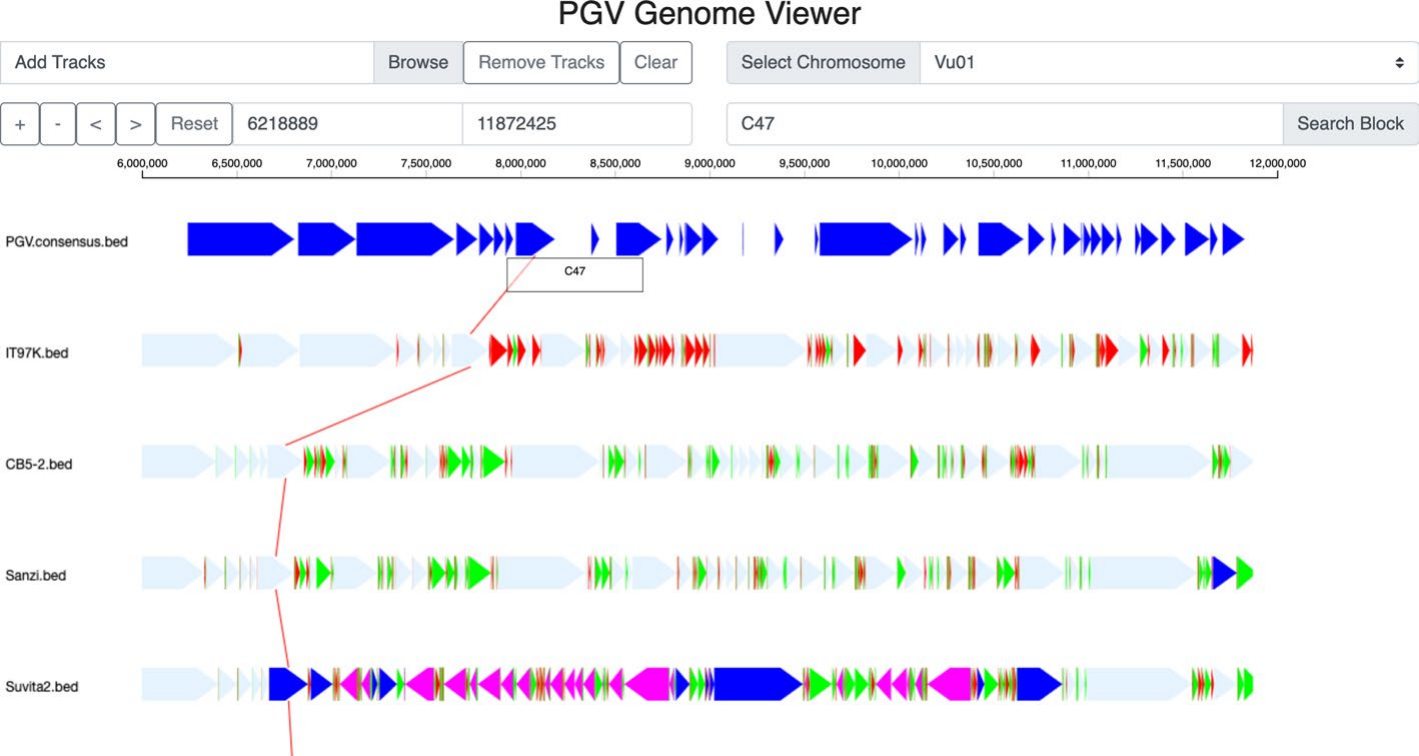
A screenshot of the PGV Genome Browser on four cowpea accessions; the first track represents the consensus ordering; IT97K, CB5-2 and Suvita2 and Sanzi are cowpea genomes; light blue blocks are core blocks with same relative ordering and orientation compared to the the consensus ordering; dark blue blocks are core blocks that are translocated when compared to the consensus ordering; pink blocks are core blocks that are inverted compared to the consensus ordering; green blocks are dispensable blocks; red blocks are unique blocks

PGV uses an efficient greedy algorithm to compute an approximation of the optimal ordering $\sigma ^*$. While the ideal outcome is to produce a single linear (consensus) ordering for each chromosome, in some situations PGV can only compute a partial ordering of the C-blocks. For example, if the input genomes contain a region which is highly variable, our greedy strategy is likely not to be able to extend the ordering across that region. For this reason, PGV maintains a set of linear orderings *O*, which is initially empty. PGV starts from an arbitrarily C-block $C_i$ which is added to *O* as the “seed” of a new linear ordering (or a path). Then, PGV determines the list $C_i$’s neighbors in the *n* input genomes and their frequency. Let $C_1$, $C_2$ and $C_3$ be the three neighbors of $C_i$ with the highest frequency, and let $f_1$, $f_2$ and $f_3$ be their frequency. If either $C_1, C_2$ or $C_3$ are already in *O*, they are not considered for the next step. Several cases are possible, (i) $f_1 \ge f_2 > f_3$, (ii) $f_1 > f_2 = f_3$, (iii) $f_1 = f_2 = f_3$. In case (i), blocks $C_1$ and $C_2$ become the candidates neighbors of $C_i$ in the consensus ordering. The consensus ordering is extended as $C_1 \rightarrow C_i \rightarrow C_2$. Then, PGV repeats the same process on $C_1$ and $C_2$, first extending to the left as much as possible, then extending to the right as much as possible. In case (ii), only block $C_1$ is added to the ordering and the process is repeated from $C_1$. In case (iii), the current consensus ordering is suspended and a new ordering starts from another arbitrary block that has not be processed yet (i.e., not in *O*).

Once PGV has processed all C-blocks, it aligns each path in *O* to the *n* genome orderings of the C-blocks to decide its orientation and determine whether it contains mis-joins. Each path and its reversed path are aligned to the original genome (C-block) orderings and an alignment score is calculated. The alignment score is +1 for an aligned block and -1 for a gap or a mismatch. The local alignment with highest score is used to determine the correct orientation and possible mis-joins. If the alignment score of the reversed path is higher than the score of the alignment for the forward path, the path is reversed. After the orientation is decided, if the overall alignment score is lower than a minimum threshold (i.e., 80% of the highest possible alignment score for that genome length), (i) the path is removed from *O*, (ii) the path is broken into two or three pieces, namely a central region with the highest alignment, a left overhang (possibly empty), and a right overhang (possibly empty), (iii) the two/three sub-paths are added to *O* and processed individually through another round of alignments. When all paths are in the correct orientations and have an overall alignments score with the input genomes of at least 80% of the maximum, PGV obtains the coordinates of each path by taking a majority vote on their best alignment against the input genomes. PGV uses these coordinates to order the paths, and produce the final consensus ordering (ideally composed of a single path). A step-by-step explanation of the algorithm using the example in Additional file 1: Figure S1 is provided in the Additional file 1.

**Fig. 2:**
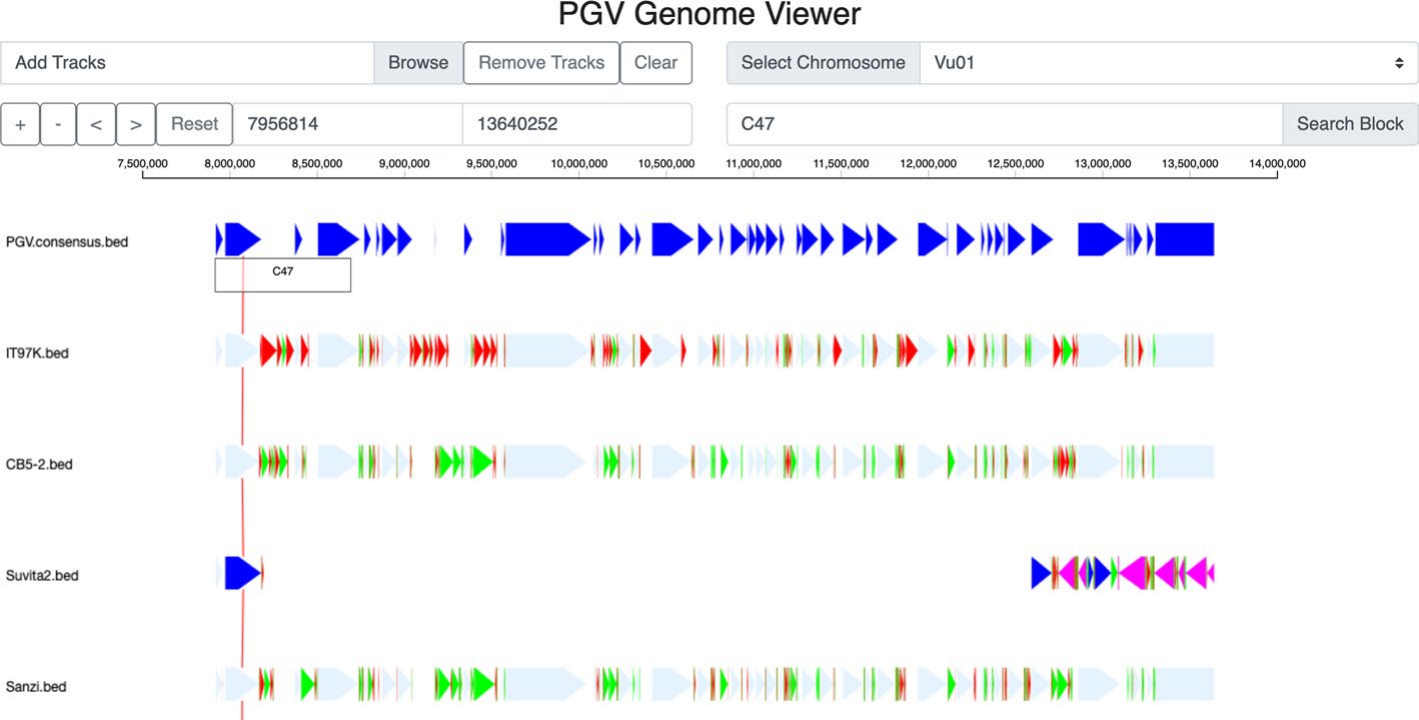
A screenshot of the PGV Genome Browser on cowpea accessions using aligned bed tracks; the first track represents the consensus ordering; IT97K, CB5-2 and Suvita2 and Sanzi are cowpea genomes; light blue blocks are core blocks with same relative ordering and orientation compared to the the consensus ordering; dark blue blocks are core blocks that are translocated compared to the consensus ordering; pink blocks are core blocks that are inverted compared to the consensus ordering; green blocks are dispensable blocks; red blocks are unique blocks

**Fig. 3:**
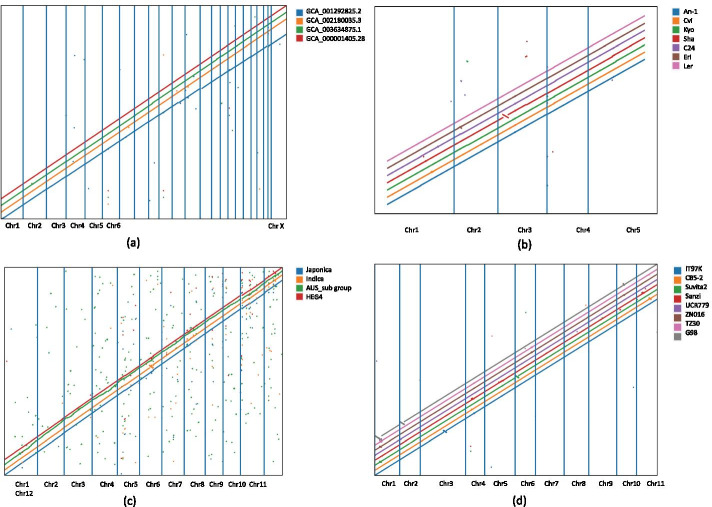
Human, arabidopsis, rice, and cowpea pan-genome analysis using PGV. The x-axis represents the coordinates of the consensus ordering of core blocks computed by PGV. Genomes coordinates for the core blocks are used on the y-axis (staggered to avoid overlapping lines)

Once the consensus ordering is computed, PGV produces a set of .bed tracks (one for each genome, plus the consensus track) that can be visualized off-line or on-line. These bed files can be processed by custom scripts to extract the coordinates of blocks of different categories. In the off-line option, PGV generates a dot-plot between the ordering of C-blocks in each genomes and the consensus ordering (e.g., see Fig. 3). This option allows users to identify major structural variations in each genome compared to the consensus ordering, and to produce figures to be shared in reports or manuscripts. The on-line option is a genome browser which allows users to visually inspect genome rearrangements (see Fig. 1). For the browser, users have the option to generate an alternative type of .bed tracks in which gaps are introduced so that C-blocks are aligned vertically (see Fig. 2). Users can upload in the browser any subset of the .bed tracks for individual genomes or the consensus ordering. Each genome is represented as a set of blocks whose sizes are proportional to the underlying sequence length. Light blue blocks are core blocks with same relative ordering and orientation compared to the consensus ordering (e.g., not reversed or translocated); dark blue blocks are core blocks that are translocated compared to the consensus ordering; pink blocks are core blocks that are inverted compared to the consensus ordering. Green blocks are dispensable blocks and red blocks are unique blocks. Tracks can be reordered by clicking on the track names and dragging them with the mouse. The usual navigation tools are available (zoom in/out, pan left/right, select a chromosome, search for a block). Clicking on a block highlights the identifier of that block, namely U for unique, D for dispensable and C for core, followed by a unique ID. Clicking on a D or C-block generates a link that connects corresponding blocks in other genomes (if they are within the current zoom window). The browser also allows users to upload GFF3 containing gene annotations, which are shown as grey blocks.

## Results

### Human

PGV was used on four *Homo sapiens* genome assemblies, namely GCA$\_$000001405.28, GCA$\_$003634875.1, GCA$\_$002180035.3, and GCA$\_$001292825.2. The multiple sequence alignment via progressiveMauve took about four days on a single-core (unfortunately progressiveMauve’s multi-core mode has been disabled by its authors); PGV took about seven minutes to find the consensus ordering. PGV identified 3548 core blocks (comprising 94.8% of the human genome), 2390 dispensable blocks and 11,807 unique blocks. Upon inspection of the initial PGV’s dot plot we determined that ten chromosomes in GCA$\_$003634875.1 were inverted. Figure 3a shows the dot-plot after reorienting those chromosomes. The four assemblies show a very high degree of consistency for the core blocks, with very few translocations indicated by the isolated dots.

### Arabidopsis

PGV was run on seven *Arabidopsis thaliana* assemblies, namely An-1 (Antwerpen, Belgium), Cvi-0 (Cape Verde Islands), Kyo (Kyoto, Japan), Sha (Shahdara, Tadjikistan), C24 (Coimbra, Portugal), Eri-1 (Eringsboda, Sweden), and Ler (Gorzów Wielkopolski, Poland) [17]. ProgressiveMauve took about six hours to compute the multiple sequence alignment (on a single-core) while PGV took about two minutes to compute the consensus. PGV identified 459 core blocks (comprising 97.43% of Arabidopsis genome), 3079 dispensable blocks and 8743 unique blocks. The higher fraction of the genome in core blocks compared to other species indicated that the seven genomes are very closely related. Figure 3b shows high consistency between the seven accessions and the consensus ordering. Observe that there is a large inversion on chromosome 3 in the Sha accession, which was also previously identified in [17].

### Rice

PGV was used on four *Oryza sativa* assemblies, namely Japonica (accession GCA$\_$001433935.1), Japonic HEG4 (accession GCA$\_$000817615.1), Indica (accession GCA$\_$002151415.1), and Aus cultivar (accession GCA$\_$001952365.3). ProgressiveMauve took about seven hours to obtain the multiple sequence alignment (on a single-core); PGV took about three minutes for the consensus analysis. PGV identified 2632 core blocks (comprising 90.11% of genome), 2531 dispensable blocks and 12,396 unique blocks. Figure 3c shows a significant amount of translocations (shown as single dots), and a (centromeric) inversion on chromosome 6 in Indica (orange anti-diagonal in the plot) which was previously reported [18].

To show the effect of introducing a more divergent genome, we repeated the experiment above by adding Indian wild rice accession GCA$\_$000576065.1 to the set. ProgressiveMauve took about nine hours to compute the multiple sequence alignment of these five rice assemblies while PGV took about three minutes to compute the consensus. PGV identified 5279 core blocks (comprising 55.66% of genome), 5729 dispensable blocks and 28,934 unique blocks. Observe that the introduction of the wild rice (1) doubled the number of all types of blocks, which indicates an increased fragmentation of the genome and (2) reduced by 35% the cumulative size of core blocks. The dot-plot analysis in Additional file 1: Figure S2 illustrates a large amount of variations in the Indian wild rice (shown as isolated green dots). Also observe in Additional file 1: Figure S2 that the consensus ordering paths are much more fragmented than in Fig. 3c.

### Cowpea

PGV was run on eight *Vigna unguiculata* genome assemblies namely IT97K [19], CB5-2, Suvita2, Sanzi, UCR779, ZN016, TZ30 and G98 (available from Phytozome CowpeaPan). Only chromosome level scaffolds were used in this experiment because unplaced contigs could introduce spurious structural variations. ProgressiveMauve took about two days to compute the multiple sequence alignment (on a single-core) while PGV took about eight minutes to build the consensus ordering. PGV detected 2863 core blocks (comprising 77.41% of the cowpea genome), 11,856 dispensable blocks and 42,484 unique blocks. Figure 3d shows several inversions (anti-diagonals): (i) two large inversions at the beginning of chromosome 1 and 2 in G98 (further analysis revealed that these two inversions were mis-assemblies), (ii) one inversion near the center of chromosome 3 of IT97K, which was previously reported by [19], (iii) an inversion shared by Suvita2, ZN016 and TZ30, previously unreported.

## Discussion

While the PGV representation can theoretically capture an arbitrarily large number of genomes, in practice the dependency from progressiveMauve limits our tool to handle up to a dozen or so of eukaryotic-sized genomes. If a more scalable/efficient multiple genome alignment tool will become available, a few changes in the preprocessing step in PGV could take advantage of it and PGV can be scaled to pan-genome with an large number of genomes.

## Conclusions

We introduced a representation of the pan-genome based on the novel notion of consensus ordering, which is reference-agnostic. Experimental results on several species demonstrate the utility of our representation. The PGV representation is reference-agnostic where it does not require one of the individual genome to be the reference. PGV can handle large eukaryotic genomes and potentially large number of genomes, and is very intuitive and simple to understand.

## Availability and requirements

- *Project name* PGV
- *Project home page* https://github.com/ucrbioinfo/PGV
- *Operating systems* MacOS, Windows, Linux
- *Programming language* Python, JavaScript
- *Other requirements* PGV can be installed with Conda, which takes care of all software dependencies
- *License* MIT
- *Any restrictions to use by non-academics* None

## Supplementary Information

**Additional file 1**. Supplementary Material includes a step-by-step description of PGV's Consensus Algorithm over a detailed example.

## Data Availability

The PGV software is available at https://github.com/ucrbioinfo/PGV. The browser can be accessed at http://pgv.cs.ucr.edu. Example BED files that can be used to test the browser can be found at the GitHub page (in the directory sample/ouputBED/). The genomes used to test PGV were downloaded from NCBI GenBank and Phytozome. Accessions for human dataset are: GCA000001405.28, GCA003634875.1, GCA002180035.3, and GCA001292825.2; accessions for rice dataset are: GCA001433935.1, GCA000817615.1, GCA002151415.1, GCA001952365.3.
