# Supplementary material for: Reference-agnostic representation and visualization of pan-genomes
Source: BMC Bioinformatics. 2021 Oct 16;22:502. doi: 10.1186/s12859-021-04424-w (PMC8520301; doi:10.1186/s12859-021-04424-w)
Supplement: Supplementary file 1 — Additional file 1. Supplementary Material includes a step-by-step description of PGV's Consensus Algorithm over a detailed example. [file 12859_2021_4424_MOESM1_ESM.pdf]

Reference-agnostic Representation and Visualization of Pan-genomes (Supplementary Materials)

Qihua Liang[1] **and Stefano Lonardi**[1,*]

[1]Department of Computer Science and Engineering, University of California, Riverside, CA 92521, USA
[*]e-mail: stelo@ucr.edu

An Example of PGV's Consensus Algorithm

In the example in Figure S1(b) we assume that PGV arbitrarily starts from $C2$, and initializes the consensus ordering $O = \{C2\}$. Then, PGV collects the frequencies for the neighbors of $C2$: $C6$ occurs four times, $C3$ occurs three times, $C1$ occurs two times, and $C5$ occurs one time. $C6$ with majority vote is added to O, resulting in $O = \{C6 \rightarrow C2\}$. In Step 2, PGV collects the frequencies for the neighbors of $C6$. In the top two, only $C5$ is not in O, and thus we extend $O = \{C5 \rightarrow C6 \rightarrow C2\}$. Similarly, in Step 4 and 5, $C4$ and $C3$ are appended to the consensus. In Step 6, $C3$ cannot be extended because both its top two neighbors are already in O. Thus PGV starts a new path by arbitrarily picking $C1$, thus now $O = \{C1, C3 \rightarrow C4 \rightarrow C5 \rightarrow C6 \rightarrow C2\}$. The top two neighbors of C_1 are H and C_2. Since C_2 is in O, only H is appended to C_1. PGV cannot extend H because H is a boundary. In Step 8, a new path is created which is extended in Step 9 to the other chromosome boundary. The preliminary set of consensus ordering is thus $O = \{p_1 = C3 \rightarrow C4 \rightarrow C5 \rightarrow C6 \rightarrow C2, p_2 = C1 \rightarrow H, p_3 = C7 \rightarrow T\}$. At this point, PGV aligns p_1, p_2 and p_3 to the individual genome orderings to decide their orientations. For instance, the alignment score of $H \rightarrow C1$ is higher than the alignment score of $C1 \rightarrow H$, so p_2 is reversed. Paths p_1 and p_3 are left as is. Then, PGV checks whether p_1, p_2 and p_3 have a good agreement with the input genomes. For instance, the alignment score of p_1 against the five input genomes is 4, 3, 5, 3, and 3, respectively. The total score for p_1 is 18, which is lower than 80% of the highest possible score, which is 0.8*5*5=20. Based on this, PGV considers p_1 not to be a good ordering and it breaks it, as follows. The highest scoring sub-path of p_1 is $C3 \rightarrow C4 \rightarrow C5 \rightarrow C6$ on the majority of the input genomes, so PGV splits p_1 into $p_4 = C3 \rightarrow C4 \rightarrow C5 \rightarrow C6$ and $p_5 = C2$, thus now $O = \{p_2, p_3, p_4, p_5\}$. PGV again checks the alignments of p_2, p_3, p_4, p_5 in O. If any of them is not sufficiently high (i.e., at least 80% of the maximum score), it will be broken again. Once this iterative process is concluded, each path in O is aligned against the genomes and the starting position of its best alignment is recorded. The position with most votes (majority) determines the coordinate of each path. For instance, the best alignment of p_4 on the input genomes are at position 4,3,4,6 and 5,

respectively. Thus, p_4 is given coordinate 4. Similarly, PGV assigns p_2 position 1, p_3 position 8 and p_5 position 3. Based on these coordinate, PGV orders the paths as $p_2 \rightarrow p_5 \rightarrow p_4 \rightarrow p_3$ which provides the final consensus ordering $H \rightarrow C_1 \rightarrow C_2 \rightarrow C_3 \rightarrow C_4 \rightarrow C_5 \rightarrow C_6 \rightarrow C7 \rightarrow T$.

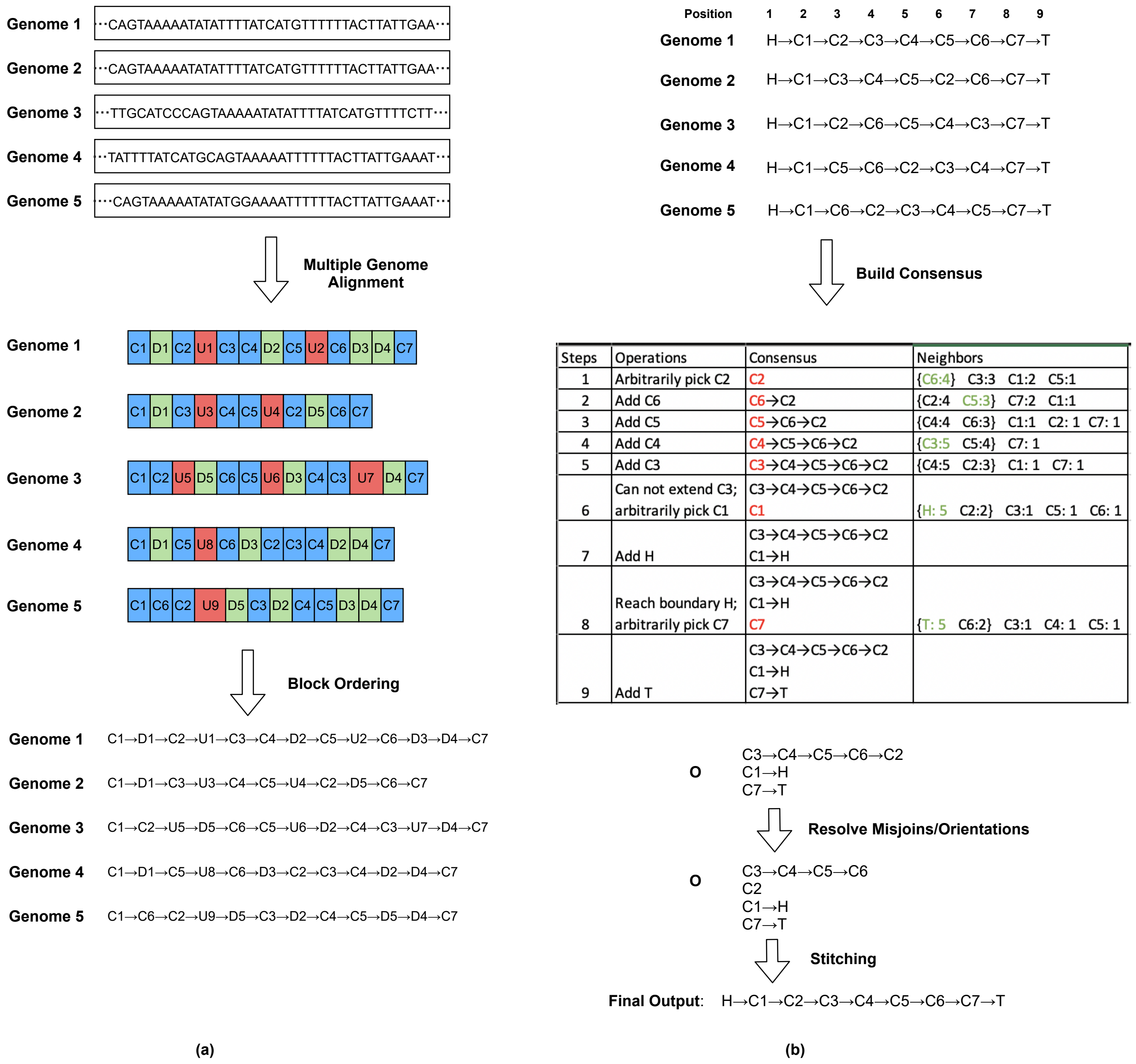

Figure S1. A detailed example of PGV's processing steps. (a) the input to PGV is a set of $n = 5$ genomes; PGV first carries out a multiple sequence alignment, then classifies each alignment block into core blocks (C), dispensable block (D) and unique block (U); each genome is then converted in an ordered sequence of C-, D-, and U-blocks, each with its corresponding identifier; (b) in the second phase, PGV computes the consensus ordering of the common blocks; red C-nodes are the active nodes; green C-nodes are the neighbors selected to be added to the linear ordering

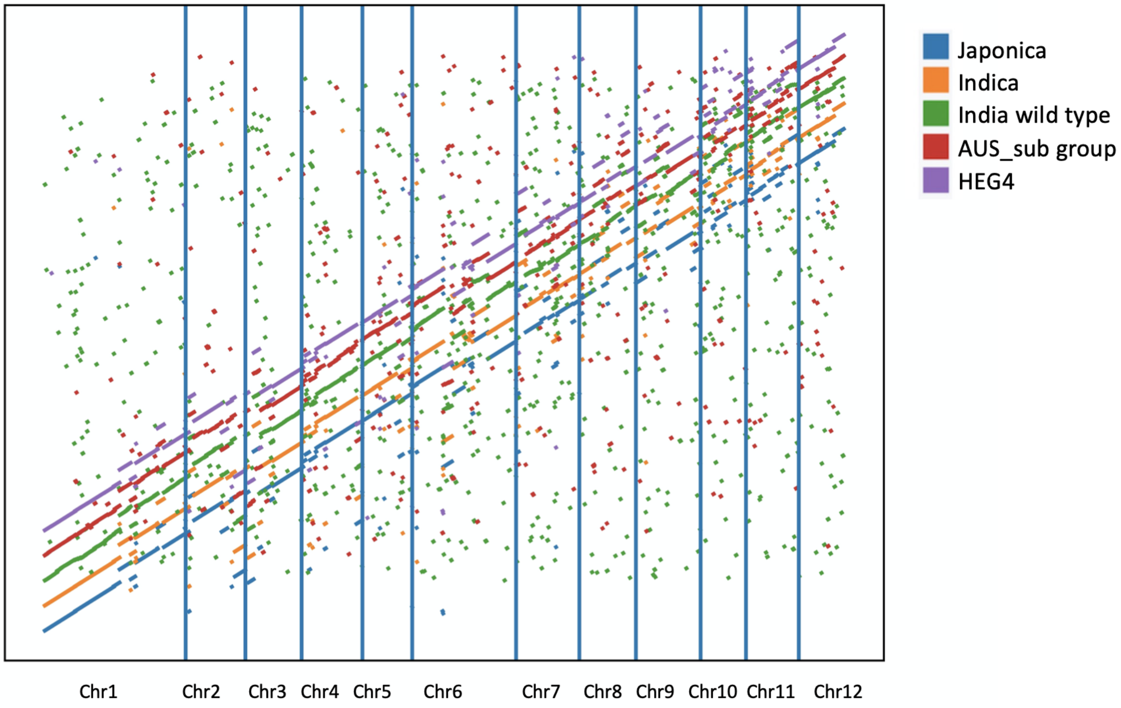

Figure S2. Rice pan-genome analysis using PGV. The x-axis represents the coordinates of the consensus ordering of core blocks computed by PGV. Genomes coordinates for the core blocks are used on the y-axis (staggered to avoid overlapping lines).
